# Tenosynovial giant cell tumour in Hoffa fat pad: a case report

**DOI:** 10.11604/pamj.2022.42.232.35027

**Published:** 2022-07-26

**Authors:** Youssef Othman, Yahia Aloui, Firas Chaouch, Saber Rabhi, Makram Zrig, Abderrazek Abid

**Affiliations:** 1 Trauma and Orthopedics Department, Fattouma Bourguiba Teaching Hospital, Faculty of Medicine, University of Monastir, Monastir, Tunisia

**Keywords:** Giant cell tumour, Hoffa fat pad, knee, case report

## Abstract

Giant cell tumour is a benign lesion classified as a fibrocystic tumour whose localization in Hoffa's fat pad is very rare. Clinical symptoms are insidious and non-specific causing a frequent confusion and delay in diagnosis therefore it should be distinguished radiologically from other similar conditions such as Hoffa´s disease and lipomas. We report a case of a 37-year-old patient, with no relevant history, who complained of a right knee pain for 5 years. Magnetic resonance imaging showed a small nodular mass in Hoffa's pad which was excised through a direct approach. Histologic examination of the specimen revealed a giant cell tenosynovial tumour. One year after surgery, the patient was asymptomatic with no local recurrence. The surgical removal of the tumour is the ideal treatment. The choice between open surgery and endoscopy depends on the site, size, and extent of the tumour.

## Introduction

Tenosynovial giant cell tumour may arise almost anywhere in the body, Hoffa´s fat pad localization is rarely seen [1]. Clinical presentation is non-specific. Knee pain is the main symptom. This tumour should be differentiated using Magnetic resonance imaging (MRI) findings from other similar conditions. We present a rare case of a painful tenosynovial giant cell tumour localized within the infrapatellar Hoffa´s fat pad and discuss the MRI finding that allow making the positive and differential diagnosis.

## Patient and observation

**Patient information:** we report a case of a 37-year-old patient, with no relevant history, who complained of right knee pain for 5 years. It was discontinuous and aggravated by strenuous activity. The patient had no sensation of the knee giving way when walking, nor did he experience any locking of his knee.

**Clinical findings:** the tof motion was limited from 0 to 110° of flexion with a moderate pain on further flexion. Neither swelling nor effusion was visible and there was no palpable mass across the joint line. There was no history of general symptoms such as night sweats, fever, loss of appetite, or weight loss.

**Figure 1:**
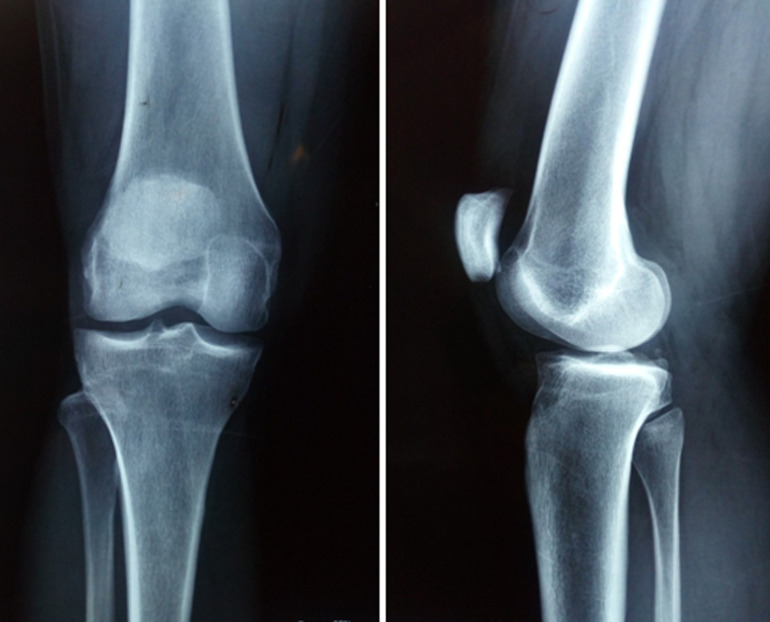
antero-posterior and lateral radiographs of the knee joint revealed no bony abnormalities

**Figure 2:**
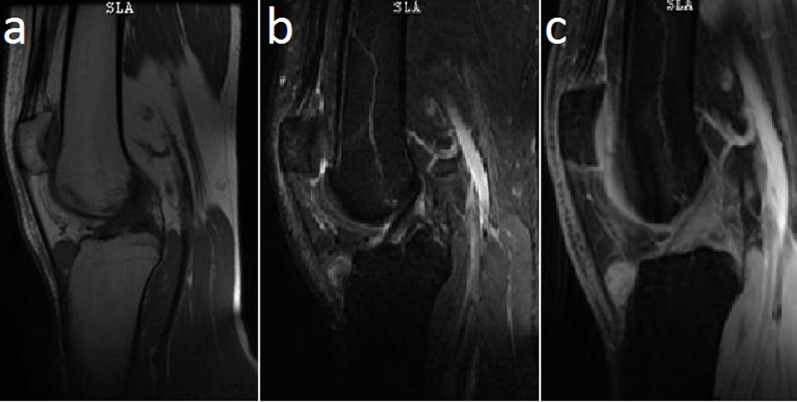
MRI showing a nodular encapsulated lesion localized in the infrapatellar Hoffa's fat pad. It presented as a hypo signal in T1 (a) and discrete T2 hyper signal interrupted by hyposignal punctuations (b) with intense homogeneous gadolinium enhancement (c)

**Diagnostic assessment:** the patient´s blood parameters were normal. Antero-posterior and lateral X-ray views showed no abnormality (Figure 1). MRI showed a limited nodular encapsulated lesion measuring 16x15mm localized in the infrapatellar Hoffa's fat pad medially lateralized. The tumour presented as a T1 hypo signal and a discrete T2 hyper signal interrupted by hyposignal punctuations with intense homogeneous enhancement after gadolinium injection (Figure 2). The bone and the patellar tendon were not interested in the lesion.

**Therapeutic interventions:** due to the localization of the tumour, we opted for open resection of the mass through a medial parapatellar approach. A 1.5 cm diameter encapsulated tumour was found. The outer surface was focally fat-laden, and the surface was yellow-brown. Therefore the mass was totally excised. Histopathological examination showed mononuclear stromal cells, osteoclastic giant cells, few lymphocytes, and macrophages. No cellular atypia or other evidence of malignancy was seen. These features allowed us to confirm the diagnosis of a giant cell tumour.

**Follow-up and outcome of interventions:** at 2 years of follow-up, the patient was asymptomatic and was able to return to his previous level of daily activities. No local recurrence of the tumour was noted.

**Patient perspective:** the patient was happy with the successful outcome of the surgery.

**Informed consent:** written informed consent was obtained from the patient for participation in our study.

## Discussion

Giant cell tumour was firstly reported by Chassaignac in 1852 as a pigmented villonodular synovitis and tenosynovitis, but recently the term of giant cell tumour is being accepted as a more accurate expression [1]. Two main varieties are recognized. The localized one affects the tendon sheath or its synovial membrane, whereas the diffuse form extensively involves the synovium. Local form usually affects tendons of the hand and foot and is usually seen in extra-articular localizations. The diffuse form, also called pigmented villonodular synovitis, is usually intra-articular and involves synovial joints, such as knees and hips [2].

Meyers *et al*. estimated the incidence for the local giant cell tumour to be 1.8 cases for each million [3]. Other investigators have reported a higher incidence of the diffuse form as opposed to the local one. Local giant cell tumour usually involves fingers and toes, such as fibroma of the tendon sheath [4]. However, the localization in the fat-pad of the knee has been reported in only seven cases [1,2,5-8] (Table 1). Our patient was a 37-year-old man. This condition usually affects individuals between the ages of 11 and 48 and women are more often interested. This tumour can cause several non-specific signs and symptoms such as locking, stiffness and giving way. Therefore, diagnosis and treatment are usually delayed for months or years such in our patient [8]. MRI is the most effective tool for the diagnosis of synovial conditions. It is helpful in diagnosing tumour-like lesions of the infrapatellar fat pad. Giant cell tumour of the Hoffa´s fat pad should be distinguished from other similar conditions such as Hoffa disease and synovial lipoma.

**Table 1:** different cases with their clinical and radiologic features, treatment and follow up

Author	Number of cases	Age	Sex	Delay	Clinical examination	MRI finding	Size	Treatment	Follow up (months )	Recurrences
Kılıçaslan *et al*.	1	32	female	6 months	Knee pain + swelling	*slightly hyperintense T1 *fat suppressed proton density; heterogeneous, high-signal intensity in the mass *Enhancement after contrast	3.5*3*1.8 cm	Open excision	18	No
Kim *et al*.	1	45	female	12 months	Knee pain + Extension limitation	*intermediate signal intensity on T1 *low to intermediate signal intensity on T2 *no enhancement of the contrast T1	2.5*3*4 cm	Endoscopic excision	12	No
Chechik *et al*.	2	48	female	12 months	Palpable swelling	*Low-intensity signal in both T1 and T2 *Diffuse enhancement after administration of gadolinium	2*2*2.5 cm	Endoscopic assisted open excision	36	No
		37	female	24 months	Swelling+ locking	*discrete, low-intensity signal mass in both T1 and T2 Diffuse enhancement after administration of gadolinium	Not available	Endoscopic assisted open excision	24	No
Panagopoulos *et al*.	1	26	male	2 weaks	Knee pain+ locking	*The mass had hypointense signal T2 and extended into the intercondylar notch.	5*4*2cm	Open excision	24	No
Abdullah *et al*.	1	11	female	24 months	Painful lump	*Encapsulated mass inferior to the patella tendon with surrounding fluid	3*3.5*1.5 cm	Open excision	35	No
Goyal *et al*.	1	33	female	3 months and half	Knee pain+ swelling	*isointensity to muscle in T1-weighted images, *low to intermediate signal intensity in T2 images, *post-contrast homogeneous enhancement in T1 weighted fat-suppressed images.	5*3*3cm	Open excision	20	No
Our case	1	37	male	60 months	Knee pain+ flexion limitation	* Hypo signal in T1 *Discrete T2 hyper signal interrupted by hyposignal punctuations *homogeneous enhancement after IV injection of GADO.	1.6*1.5 cm	Open excision	24	No

Lipomas are seen on MRI as a typical discrete lipomatous mass with hyperintensity on both T1- and T2- weighted images with the loss of signal on appropriate fat-suppressed sequences. Hoffa´s disease is an intrinsic disease of Hoffa´s fat pad firstly described in 1904 by Albert Hoffa as an acute or chronic inflammation of the infrapatellar fat pad [9]. Its pathophysiology is not well known. Typical MRI features of Hoffa´s disease are observed in Sagittal T1- and proton density Fat Sat-weighted sequences. It shows a diffuse oedema-type signal (hypointense on T1-weighted images and hyperintense on PD fat sat sequences) within the Hoffa´s fat [9].

In the case of a giant cell tumour, MRI shows T1 hypointense and a discrete T2 hyperintense signal with gadolinium homogeneous enhancement. This lesion is characterized by hypointense punctuations best seen on fast field echo sequence MRI images suggesting the presence of hemosiderin deposits that are typical of this condition. This sequence was not used to establish the diagnosis in our case and even in the majority of reported cases. The suitable treatment of the local form is controversial due to the lack of evidence and the high risk of recurrence. The local Hoffa´s fat extra-articular form of giant cell tumour can be removed by open surgery [2,6-8], endoscopic procedure [1], or endoscopic assisted open excision [5]. In our case, the patient underwent an open complete resection of the mass since it is an extra-articular and accessible tumour. The endoscopic approach is a well-validated alternative that could be used in some selected patients. However, open surgery is preferred in order to provide a complete excision and therefore reduce recurrences [5,6]. For recurrent and refractory disease, adjuvant therapy such as external beam radiotherapy should be considered. Furthermore, denosumab which is now a well-recognized treatment for bone giant cell tumours is now tested by some studies for soft tissue giant cell tumours [10].

## Conclusion

Although tenosynovial giant cell tumour has a rare occurrence in Hoffa's fat pad, it should be considered as a potential cause of non-traumatic knee pain. The presence of hemosiderin deposits in MRI has best seen, especially on fast field echo sequence is strongly suggestive of the diagnosis and permits to eliminate of differential diagnoses such as lipomas and Hoffa´s disease. The diagnosis is only confirmed by histopathological examination. Open surgical resection is preferred in order to ensure complete resection and to avoid recurrences.
